# Dynamics of Neurogenic Signals as Biological Switchers of Brain Plasticity

**DOI:** 10.1007/s12015-024-10788-2

**Published:** 2024-09-11

**Authors:** João F. Moreira, Susana Solá

**Affiliations:** Research Institute for Medicines (iMed.ULisboa), Faculty of Pharmacy, Universidade de Lisboa, Av. Prof. Gama Pinto, 1649-003 Lisbon, Portugal

**Keywords:** Autocrine, Neural Stem Cell, Neurogenic Niches, Paracrine, Signals

## Abstract

The discovery of adult neurogenesis in the middle of the past century is considered one of the most important breakthroughs in neuroscience. Despite its controversial nature, this discovery shaped our concept of neural plasticity, revolutionizing the way we look at our brains. In fact, after the discovery of adult neurogenesis, we started to consider the brain as something even more dynamic and highly adaptable. In neurogenic niches, adult neurogenesis is supported by neural stem cells (NSCs). These cells possess a unique set of characteristics such as being quiescent for long periods while actively sensing and reacting to their surroundings to influence a multitude of processes, including the generation of new neurons and glial cells. Therefore, NSCs can be viewed as sentinels to our brain’s homeostasis, being able to replace damaged cells and simultaneously secrete numerous factors that restore regular brain function. In addition, it is becoming increasingly evident that NSCs play a central role in memory formation and consolidation. In this review, we will dissect how NSCs influence their surroundings through paracrine and autocrine types of action. We will also depict the mechanism of action of each factor. Finally, we will describe how NSCs integrate different and often opposing signals to guide their fate.

**Graphical Abstract:**

Different signaling pathways responsible for signal integration of NSCs-secreted autocrine/paracrine signals: Numerous superficial receptors are stimulated upon contact with NSCs-secreted factors. Interestingly, this schematic representation of the different pathways shows how different signals often converge into the same pathway. This allows the NSC to adopt the correct behavior in response to external stimuli.

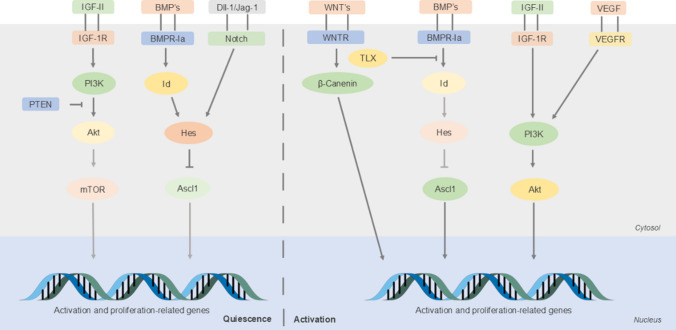

## Introduction

Adult neurogenesis has always been a controversial subject amongst neuroscientists [1]. In 1928, Santiago Ramon y Cajal, considered by many as the father of neuroscience, stated *“In the adult centers, the neural paths are something fixed and immutable: everything may die, nothing may be regenerated”*. This led to the generalized belief that adult neurogenesis did not occur. Consequentially, the discovery of adult neurogenesis by Josep Altman [2] was met with skepticism and widely ignored. It was only in the 1990s that Altman’s findings were backed by reports of new neurons in the hippocampal dentate gyrus subgranular zone (SGZ) [3], and subventricular zone (SVZ) of adult rodent brains [4]. A year later, Eriksson et al. 1998 used a thymidine analogue and NeuN to demonstrate human adult neurogenesis [5]. Despite these studies supporting adult neurogenesis in humans, a recent study by Sorrels et al. 2018 [6] reported this process drops sharply within the first year of life to nearly undetectable levels after 7 years. Surprisingly, a year later, a study by Boldrini et al. 2018 [7] contradicted Sorrels’ results stating the existence of adult neurogenesis in the human brain throughout our entire lifespan. One possible explanation for these contradictory results is the different methodologies used. While Sorrelis [6] used tissue from people with diagnosed chronic epilepsy, Boldrini [7] chose tissue from people with no diagnosed psychiatric disorders. Moreover, other group has recently reported data supporting Boldrini’s work [8]. Diversity of findings, as well as methodological differences, delay consensus in the field of adult neurogenesis in humans. However, nowadays it is assumed that this process mainly happens in two regions or neurogenic niches: the SGZ in the dentate gyrus of the hippocampus and the SVZ [9].

Several factors differentiate SVZ from the SGZ neurogenic niches, such as different anatomic locations, distinct surroundings and their proposed relevance after maturation. Although the neurogenesis process begins with the activation of quiescent NSCs in both niches [10], one major difference is the type of neurons generated by NSCs.

In the SGZ, neurogenesis has four fundamental stages: the precursor, the early survival, the post-mitotic and the late maturation stages. At the beginning of the process type 1 NSCs become activated and originate type 2 cells, which characteristically show high proliferative capacity. Type 2 cells eventually differentiate into type 3 cells, which are less proliferative. This marks the beginning of the early survival phase, with a concomitant shift from high proliferation to high apoptotic cell death. As a consequence, there is a stabilization of the NSC pool and, subsequently of their differentiation. After this point, neurons begin to integrate into the pre-existing hippocampal networks (post-mitotic phase) and, over time, acquire the electrophysiological characteristics of fully mature hippocampal neurons (late maturation phase) [11].

Unlike hippocampal neurogenesis, the neurogenic process in the SVZ can be divided into three fundamental stages: the precursor, the migratory and the maturation stages. Similarly to what occurs in the hippocampus, the process starts when type B NSCs become activated and give rise to type C transit-amplifying cells. These cells further proliferate and then differentiate into type A neuroblasts. The differentiation into neuroblasts marks the end of the precursor cell phase and the beginning of the migratory phase, in which neuroblasts migrate from the SVZ to the olfactory bulb via the rostral migratory system (RMS). Once they reach the olfactory bulb, the migratory phase ends and the maturation phase begins with the surviving neurons being integrated as GABAergic interneurons [12].

**Fig. 1:**
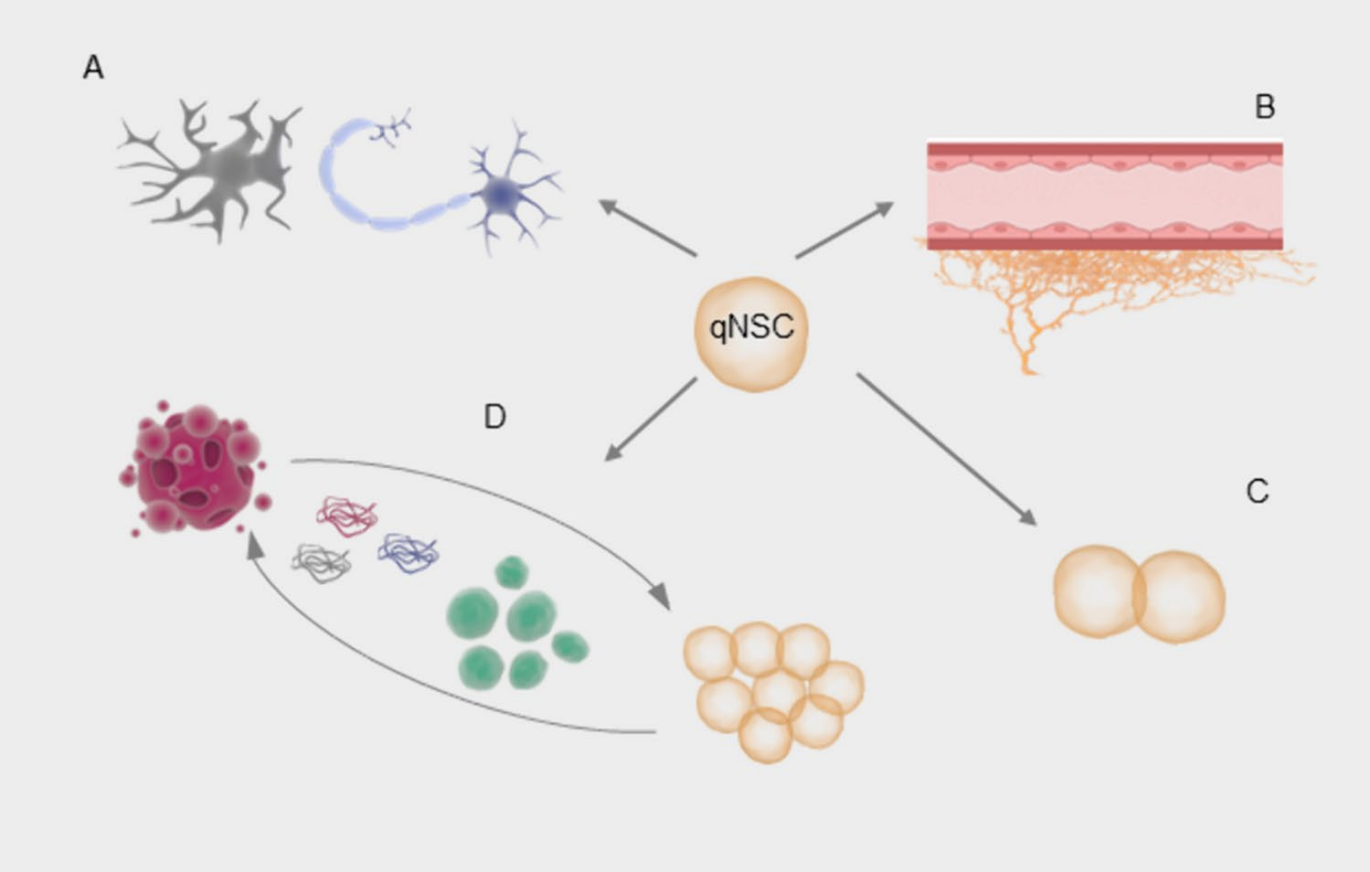
Different roles of adult NSCs in the neurogenic niche: Schematic representation of the various roles adult NSCs play in the neurogenic niche. A- NSCs possess the ability to differentiate into different cell types such as glutamatergic and GABAergic neurons; B- they are uniquely positioned in the niche to be in close contact with the vasculature; C- when quiescent, they express several genes that enhance cell-to-cell communication; D- they are constantly monitoring and reacting to changes in their surroundings, often promoting homeostasis through the release of soluble factors or EV’s

Noteworthy, the entire process of neurogenesis is supported by NSCs, multipotent cells capable of differentiating into various neuronal cell types (Fig. 1A) and sustaining their population through self-renewal. Despite having great replicative potential, one of the hallmarks of NSCs is the ability to remain in a quiescent state for long periods, rendering them resistant to metabolic stress and genetically stable [13]. As a result, their energy consumption is low, their proliferative potential is preserved, and there is less damage accumulation in their proteins, DNA and mitochondria, preventing senescence or malignant transformation [14].

Despite their time spent in a quiescent state, these cells are involved in an array of processes. Curiously, a recent study showed upregulation of receptors in quiescent NSCs which are usually downregulated during their activation [15]. This might be a consequence of epigenetic changes. In fact, several transcriptome analyses showed that epigenetic changes of NSC activation are not restricted to cell cycle-related genes. For example, active NSCs present higher expression of DNA repair-, transcription- and translation-related genes, while quiescent NSCs appear to express more genes related to cell signaling, cell-cell communication (Fig. 1C), extracellular matrix and cell adhesion processes [16, 17]. These findings suggest that, during quiescence, NSCs are actively “listening” and reacting to their surroundings. Indeed, quiescent NSCs reside in two highly specialized and dynamic microenvironments called niches, capable of anatomically housing NSCs while influencing their development. In these areas, they are in the perfect location to receive extrinsic cues from both the surroundings and the rest of the body and relay them to the members of the neurogenic niches [9]. These features allow NSCs to act as sentinels of neuronal homeostasis by constantly receiving and releasing extravascular vesicles and factors in response to environmental changes (Fig. 1D).

Interestingly, NSCs derived from SGZ and SVZ are in close contact with the vasculature through their dendrites and axons, respectively [18]. The vasculature in both of these regions has specific characteristics that enhance their exposure to systemic cues and render them capable of sensing systemic cues, such as higher permeability and slower blood flow (Fig. 1B) [19]. On the other hand, NSCs from both niches contact with a variety of cell types in their intermediate domains, exerting and receiving a profound influence from their progeny, mature neurons, astrocytes, and even the immune system through microglia [18, 20]. Finally, in their proximal domain, NSCs in the SVZ niche contact directly with the cerebrospinal fluid, namely through specialized cilium at the end of their axons which in turn are surrounded by ependymal cells in a pinwheel-like structure [18]. NSCs in the SGZ niche, in turn, extend their axons to the granular layer of the dentate gyrus, where they receive GABAergic and glutamatergic influence from hilar interneurons and Mossy cells respectively [21].

Indeed, due to their structural differences, NSCs in the SVZ are more prone to be influenced by systemic signals—either from the CSF or from the blood—than SGZ-derived NSCs. Hippocampal NSCs, in turn, have more intimate contact with neurons from existing neural networks at their intermediate and distal domains, rendering them susceptible to being modulated by neurotransmitters such as GABA and Glutamate. Despite these differences, both angiogenesis and neurogenesis often occur in parallel in the SGZ, but not in the SVZ [22], suggesting a more profound relationship between the vasculature and type 1 cells than with their type B counterparts. Interestingly, NSCs from the SVZ remain in close contact with their progeny until the end of the precursor phase. This period is characterized by intense communication between both cell types through several feedback mechanisms further described. These processes are partially responsible for defining the neurogenesis rate in this niche [18].

Together, these statements have strongly supported the idea that even in long quiescent periods, NSCs play a pivotal role in brain homeostasis, upregulating the expression of proteins, sensing the health of the body and acting as powerful sentinels in the brain. As they respond to autocrine, paracrine and endocrine types of action, they also influence neuroplasticity in the adult brain through the same type of action. Here, we will emphasize some autocrine/paracrine factors secreted by NSCs and their contribution to homeostasis maintenance in the brain.

## Wnts Signals

The Wnt family of proteins, is involved in a multitude of processes [23]. In the context of NSCs, they have been mainly related to self-renewal [24] and neuronal progenitor cell (NPC) proliferation [25]. However, numerous studies have also demonstrated that this pathway also plays a key role in the differentiation of NPCs [26, 27]. The influence of Wnts in this process may depend on different factors, including the type of Wnt, environmental cues [28] as well as cell-intrinsic proprieties. Strikingly, it has been shown that Wnts secreted by both SGZ- and SVZ-derived NSCs bind to their specific receptors and inhibit the phosphorylation and degradation of β-catenin resulting in an autocrine type of action [24]. This effect is mediated by the glycogen synthase kinase 3β [29] and leads to β-catenin accumulation and nuclear translocation to activate several targeted genes such as *Akt* (protein kinase B) [24, 30]. Indeed, the Akt pathway plays a key role in signal integration, promoting an inter-pathway cross-talk between Wnts, IGF-II and others leading to the proliferation and self-renewal of NSCs. The effects that result from β-catenin accumulation in the cytoplasm seem to partially depend on the concomitant presence of the fibroblast growth factor 2 (FGF2) signaling. In fact, in the presence of FGF2, high levels of β-catenin result in the preservation of a proliferative state, while, in the absence of FGF2, this same phenomenon leads to neuronal differentiation [31]. Another important factor that deeply influences Wnt’s actions is the apparent feedback loop between the NSC-secreted Wnts and the NPC-secreted Wnt inhibitor dickkopf-related protein 1 (DKK1) [32]. Since Wnts promote the proliferation of NPCs, as the quantity of Wnt secreted increases, the number of NPCs also increases, leading to a higher secretion rate of DKK1, and therefore to a repression of the excessive Wnt expression which could lead to an NSC pool depletion. This poses a feedback mechanism between NSCs and their progeny as they control the number of each other. More importantly, it assures the maintenance of both the NSC and NPC pool if there is a need for increased neurogenesis. Nevertheless, this regulatory process might be context-dependent as it is influenced by the presence of other factors such as FGF. Indeed, the choice between proliferation and differentiation is not dependent on the influence of one single factor but rather on a favorable context.

The central role of Wnts in neurogenesis and brain homeostasis is becoming more evident. Under homeostatic conditions, this pathway is key in promoting NSC proliferation or differentiation in a context-dependent mode of action [33]. Besides regulating neurogenesis, the Wnt pathway is proposed to play a role in the age-associated decrease in neurogenesis. Several studies have reported a decrease in Wnt secretion with aging along with a concomitant increase in the secretion of Wnt inhibitors, such as DKK1 [34, 35]. Wnt deregulation is also associated with dementia-related pathological states such as Alzheimer’s disease (AD). In fact, an altered expression of key genes that control the Wnt pathway was found in AD patients. Among these, increased levels of Wnt inhibitors such as DKK-1 [36], glycogen synthase kinase 3β [37] and altered expression of *Wnt inhibitory factor 1* [38] are proposed to contribute to the neurogenesis impairment observed in AD. Therefore, Wnts seem to be attractive targets to design a therapeutical strategy to cope with some cognitive deficits provoked by AD. However, there is still a long way to go since the role of neurogenesis in AD is not well clarified.

## Neurotransmitter Signals

To work properly, neurons must communicate through synapses, more specifically, through the release of a group of chemical messengers generically referred to as neurotransmitters. Examples of those molecules include serotonin, dopamine, GABA, and glutamate. NSCs in the SGZ turn into glutamatergic hippocampal neurons after maturation and are key players in processes such as memory consolidation, mood regulation and even protection from glutamate excitotoxicity. Several studies have shown glutamate influences NSC behavior, triggering self-renewal divisions [39, 40]. This action is mediated not by glutamatergic receptors, but rather by the family of excitatory amino acid transporters (EAATs), more specifically by EAAT1, which is expressed in immature NSCs [41]. It has been shown that cellular import of glutamate via EAAT1 results in the alteration of the expression of several genes which ultimately leads to higher expression of fatty acid synthetase (FASN). The activity of this enzyme thrives lipogenesis, a process that is key to NSC proliferation and self-renewal [42]. Moreover, a recent study showed that deletion of EAAT1 resulted not only in the reduction of the NSC number but also in the reduction of neurogenesis [41]. This suggests that EAAT1 ablation does not result in quiescence exit and higher differentiation rate but rather in a slow decline of NSC and neurogenesis, mimicking what happens during aging. Through this process, fully mature SGZ-derived NSCs contribute to the conservation of an adequate NSC pool through a paracrine type of action [41].

In addition to glutamate, other neurotransmitters play a pivotal role in NSC dynamics. One example is the gamma-aminobutyric acid (GABA). This molecule is considered the main inhibitory neurotransmitter in the brain. It is responsible, along with glutamate, for the maintenance of a homeostatic excitatory/inhibitory balance [43]. Besides playing a key role in establishing an appropriate excitatory/inhibitory balance in the brain, GABA was also shown to influence NSC proliferation. Particularly in the SVZ region, where several studies have not only demonstrated the influence of GABA in NSC fate *per se*, but also the effects of acute and chronic GABA_A_ receptor agonist treatment. Both *in vitro* and *in vivo* studies showed that GABA agonists led to decreased proliferation potential of NSCs through a mechanism dependent on H2AX histone, while treatment with a GABA_A_ receptor antagonist had the opposite result [44]. Additionally, neuroblast-secreted GABA influences neurogenesis by reducing the proliferation of NSCs at early stages of maturation [45].

Furthermore, NSCs and their progeny can also influence GABA activity through the endozepine diazepam binding inhibitor (DBI). This small protein is highly expressed in the SVZ and rostral migratory system and, by binding to part of GABA_A_ receptors, dampens GABA activity and induces cell proliferation *in vivo* [46, 47]. The delicate balance between pro-proliferative DBI and anti-proliferative GABA is controlled by NSCs in different stages of differentiation. The source of GABA can be traced to neuroblasts [45, 48], while DBI is mainly produced by NSCs and NPCs but not by neuroblasts or neurons [46]. Thus, neuroblasts inhibit NSC and NPC proliferation through GABA, while NSC and NPC promote their proliferation through DBI [46]. Importantly, these opposite, yet complementary, paracrine actions form a feedback mechanism between NSCs and their progeny that assures an adequate balance between proliferation and differentiation.

These feats shed some light on the unique nature by which neurotransmitters affect NSC behavior. Contrary to other factors described here, whose actions often depend on signaling pathway integration, the nature of the dominant cell type in the niche promotes either a glutamatergic or a GABAergic context that defines the type of feedback between NSCs and their progeny.

Given the relevance of an adequate excitatory/inhibitory balance in normal brain physiology, both glutamate and GABA are considered important players in normal brain function. Glutamate is the main neurotransmitter in the brain, being responsible for sensory processing, learning and emotional responses. Interestingly, low glutamate levels enhance stress-coping responses that include increased hippocampal neurogenesis. However, long-term exposition to stress leads to higher glucocorticoid levels, which in turn cause excessive glutamate signaling. This process occurs in parallel with decreased GABAergic and serotonergic activity, indicating depression [49] and often resulting in hippocampal neurotoxicity and reduced DGZ neurogenesis [50]. Curiously, the actions of fluoxetine and physical exercise, two known antidepressants, depend in part on the regulation of glutamatergic activity. Fluoxetine, a selective serotonin reuptake inhibitor, enhances serotonergic transmission thereby ameliorating glutamatergic activity. Exercise, in turn, decreases stress and cortisol levels, leading to downstream inhibition of glutamate. The increased hippocampal neurogenesis promoted by both treatments [50], partially depends on this process [50, 51]. Another interesting aspect of depression is the recurrent use of benzodiazepines to treat anxiety. These molecules act by binding to GABA receptors and promoting their activation in a similar mode to GABA. If in the short-term they are highly effective in treating depression-associated anxiety, we can hypothesize that their GABA-like activity reduces neurogenesis by a mechanism similar to GABA. This raises the question of whether by treating anxiety recurrently with benzodiazepines we are not diminishing neurogenesis and consequentially preventing a physiologic mechanism capable of ameliorating depression. Indeed, a study by Boldrini et al. 2014 [52] showed that benzodiazepine treatment prevents fluoxetine-induced neurogenesis, and that excessive GABAergic activity in the DG causes neuronal apoptosis.

## The MCFD2 Signal

The multiple coagulation factor deficiency 2 (MCFD2), or stem cell-derived neural progenitor cell supporting factor (SDNSF), as initially described by Toda et al. 2003, has been historically associated with a coagulation deficiency. Curiously, besides playing a key role in the coagulation process, its genetic sequence suggests MCFD2 might influence other processes than coagulation [53]. Interestingly, the release of MCFD2 in the brain is restricted to a small number of cell types in the dentate gyrus of the hippocampus, including NSCs. Since this area is a major neurogenic niche, it has been suggested that MCFD2 might influence the neurogenesis process. In fact, MCFD2 expression was found upregulated in the hippocampus after neurogenesis stimulation *in vivo*, corroborating a possible link between MCFD2 and neurogenesis. Furthermore, Toda et al. also found that exposure of NSCs to MCFD2 enhanced NSC survival, while also maintaining the self-renewal potential and multipotency of these cells [54]. These findings were then confirmed by other studies demonstrating that MCFD2 promotes an undifferentiated state in NSCs similar to those observed with FGF2, indicating that both of these factors might influence the same signaling pathways. In fact, treatment with FGF2 or MCFD2 resulted in lower levels of phosphorylated c-Jun N-terminal kinases (JNK) and extracellular signal-regulated kinase (ERK), ultimately translating into mitogen-activated protein kinase (MAPK) signaling pathway inhibition [53]. Of note, the MAPK pathway plays a central role in the integration of signals from cytokines and other factors into intracellular events through receptor tyrosine kinases, ultimately influencing several processes, such as proliferation and survival. In addition to MAPK, MCFD2 also inhibits the Akt pathway [55].

These findings suggest that MCFD2 not only triggers an undifferentiated state in the NSCs but also enhances their self-renewal. These effects seem to reflect an autocrine type of action once hippocampal NSCs are also the most likely source of MCFD2. Interestingly, the expression of MCFD2 was shown to be increased when neurogenesis is stimulated. Therefore, we might consider MCFD2 as a homeostatic “brake” signal of neurogenesis to prevent excessive differentiation of NSCs. This effect is likely mediated by the inhibition of the Akt pathway, which is critical for signaling integration in proliferation. Unfortunately, the role of MCFD2 in neurogenesis and brain physiology is not well understood. Additional studies should be performed regarding the specificity and the temporal signaling proprieties of MCFD2, and many important questions remain to be answered. Does MCFD2 still exert excessive homeostatic “brake” activity upon disease? Are MCFD2 expression levels constant throughout life? Does MCFD2 have a similar type of action in the SVZ?

## The IGF and VEGF Signals

Insulin-like growth factors I (IGF-I) and II (IGF-II) belong to a system of factors often designated as “IGF system”, whose main receptor is the insulin-like growth factor receptor 1 (IGF-1R) [56]. The binding of IGF-II to IGF-1R, which is expressed widely in the brain [57], triggers downstream activation of cell proliferation-related MAPK and phosphoinositide 3-kinase (PI3K) -Akt—forkhead box (FOXO) pathways [58, 59]. Curiously, these signaling pathways are reported to induce either proliferation or differentiation in a context-dependent manner [60]. One possibility of such context-dependent interaction might be the presence of other proliferative signals whose actions are Akt-dependent, including Wnts or pleiotrophin protein (PTN).

The IGF-II signal also binds to insulin-like growth factor receptor 2 (IGF-2R) promoting memorization and learning consolidation [56, 61]. Considering the role of IGF-II in NSC homeostasis, Ziegler et al. studied the influence of *IGF-II* deletion on NSCs. As expected, inhibition of *IGF-II* resulted in fewer NSCs in both the SVZ and SGZ, which resulted in learning, memory and stress-coping abilities impairments. In addition, the ablation of IGF-II also led to higher NSC differentiation in the SVZ areas, resulting in increased inhibitory GABAergic interneurons in the olfactory bulb and olfactory deficits [62].

One of the most prominent effects of IGF-II is its role in memory formation and consolidation. This is, in part, a consequence of the higher NSC proliferation and differentiation rates in the presence of IGF-II. Interestingly, hippocampal NSCs are also a source of IGF-II [63–65], showing their role in neurogenesis regulation and, consequentially, neuroplasticity. This process also reflects an autocrine type of action in the SGZ. Notably, abnormalities in the IGF-II signaling have been associated with neurological diseases such as Alzheimer’s and Parkinson’s diseases [60]. These findings suggest a possible relationship between loss of homeostasis in the neurogenic niche and the presence of pathological states. However, this association is yet to be established. Is this type of disease the result of a more susceptible brain due to age-associated neurogenesis decline? Or is it possible that the neurodegenerative disease-associated deregulation of neurogenesis is the main cause behind the observed cognitive deficits? Interestingly, some studies in animal models of diseases such as AD, Parkinson’s and Huntington’s disease reveal that running, a known inducer of IGF-II, leads to modest improvements in some cognitive deficits, suggesting a possible role for neurogenesis.

In the adult mammalian brain, neurogenesis has been shown to occur near growing blood vessels [22]. This finding makes the vasculature a suitable candidate as a provider of supporting factors that regulate the neurogenic niche [66]. One of the factors that participate in this regulation is the vascular endothelial growth factor (VEGF). The VEGF signal is secreted by endothelial cells in the vasculature and can exert its influence as a neurogenic factor [67], increasing NSC proliferation through the activation of the PI3K-Akt pathway [68]. Interestingly, as the IGF factor, the expression of VEGF also increases in response to various pro-neurogenic factors such as exercise [69] and environmental enrichment [70], being crucial for the therapeutic effects of antidepressants [71].

Curiously, besides endothelial cells, the progeny NSCs are additional sources of VEGF in the SGZ. Notably, these cells were found to secrete relatively high amounts of VEGF, suggesting an autocrine type of action. For example, in the hippocampal neurogenic niche, undifferentiated NSCs are responsible for almost 30% of the VEGF present in that region. Strikingly, although inhibition of NSC-derived VEGF resulted in an initial increase in NSC proliferation and differentiation, prolonged inhibition of this factor significantly repressed neurogenesis [72]. These findings may appear contradictory to the effects of the VEGF signal. Still, they suggest an alternative role for the VEGF. In homeostatic conditions, the combined production of VEGF from NPCs and other cell types appears to be sufficient to induce VEGF-driven cell proliferation and NSC self-renewal over differentiation [73]. In contrast, when the NSC production of VEGF is inhibited, NSCs proliferate less and differentiate faster, resulting in an initial increase in neurogenesis but at the cost of NSC pool depletion.

Interestingly, higher circulating lactate levels caused by exercise are related to higher VEGF expression in the brain [74]. Indeed, the association between exercise practice and increased neurogenesis is well-established and is generally traced back to higher levels of neurogenic peptides such as VEGF or brain-derived neurotrophic factor. Considering NSCs are perfectly positioned to receive systemic signals from the vasculature, it could be hypothesized that NSCs play a crucial role in mediating exercise-related neurogenesis through increased VEGF secretion. This mechanism may also be responsible for some of the harmful effects of obesity and depression on neurogenesis. As it happens with exercise, these conditions affect the composition of systemic cues in the blood. As a result, the secretome of NSCs may reflect some of these changes. Thus, depression or diet-related decreased secretion of VEGF could be responsible for NSC pool exhaustion and neurogenesis impairment.

## The BMP and Noggin Signals

An additional important feature of NSC quiescence is the delicate balance between bone morphogenetic protein (BMP) and its antagonist Noggin. The BMP is part of the superfamily of cytokines transforming growth factor-β (TGF-β) and has a variety of effects on the nervous system, influencing processes from neurodevelopment to neurogenesis, gliogenesis and even apoptosis [75, 76]. It has been shown that BMP signaling is more activated in quiescent NSC and NPC in the SGZ neurogenic niche, remaining inactivated in proliferating cells. Moreover, treatment of NSC with BMPs was shown to reduce cell proliferation and induce quiescence. On the other hand, blockage of BMP through its antagonist Noggin resulted in NSC activation and quiescence exit. It initially promoted neurogenesis, however, ultimately resulted in NSC pool exhaustion [77].

BMP is also expressed and secreted by NSCs and their progeny in the SVZ neurogenic niche, but not by neuroblasts, while its antagonist, Noggin, is secreted by ependymal cells [78]. In the SVZ, the BMP signaling inhibits neuronal differentiation, while promoting an astroglial commitment [78]. The BMP binds to its receptor BMPR-Ia, phosphorylating the suppressor of mothers against decapentaplegic 1 (SMAD1) protein and triggering the canonical BMP pathway [77]. This, in turn, was found to upregulate the anti-differentiation genes *Id 1–4* and *hairy and enhancer of split 1 (Hes1)* and diminish the number of NSC divisions [79]. Indeed, experiments with human fibroblasts showed deletion of *Hes1* results in inability to resume proliferation after induction of quiescence, indicating that *Hes1* is key to maintaining the reversibility of NSC quiescence [80]. In addition, it has been shown that the expression of *Hes1* can partially depend on the activation of the Notch pathway [81], showing that both quiescence-promoting pathways can synergistically influence each other.

All in all, the outcomes of the BMP signaling are highly context-dependent since in the SGZ neurogenic niche they enhance NSC quiescence and in the SVZ lead to astroglial lineage commitment. However, in both contexts, it is unequivocal that BMP signaling decreases neurogenesis in an autocrine type of action. Many factors influence the actions of both noggin and BMPs. It thus appears that a fine-tuned balance between the pro-neurogenic noggin and the pro-quiescence BMPs is, in part, responsible for the maintenance of homeostatic conditions [82]. Running, a known inducer of hippocampal neurogenesis, enhances noggin while repressing BMP signaling. Strikingly, the neurogenic effects of running were blocked upon higher expression of BMP [83]. In addition to running, BMP seems to be related to AD disease. Higher levels of BMPs along with neurogenesis impairments were observed in an animal model of AD, the later rescued by noggin [84, 85].

## The PTN Signal

The PTN was discovered in the 90’s and its name originates from the word pleiotropy, a term that describes multiple effects, therefore influencing different cellular processes [86]. Hence, pleiotrophin actions differ with tissue and context. For this reason, it is without surprise that PTN plays important roles in processes such as adipocyte differentiation [87], bone development [88] and mammary epithelial cell differentiation [89]. However, the most studied role of PTN has been in neurodevelopment.

PTN is widely produced in the brain by pericytes and it is essential for normal neuronal functioning [90], as well as oligodendrocyte differentiation [91]. Other sources of PTN in the brain are the NSCs located in SGZ. Notably, the removal of PTN in the hippocampal regions leads to shorter and less complex dendrites and lower spine density, resulting in poorer integration of newborn neurons into the existing hippocampal network. As a consequence, mice with inhibited PTN expression showed hippocampal learning deficits and worse performances in both fear conditioning and novel objective localization tests. These effects were found to be anaplastic lymphoma kinase (ALK)-Akt-dependent, since the pharmacological inhibition of this PTN receptor resulted in smaller and simpler dendrites. Notably, both ALK knockdown and ablation of Akt had the same effect even in the presence of an exogenous PTN treatment [92].

Curiously, similarly to Wnts, there is an age-associated decline expression of hippocampal PTN. This suggests that NSCs become increasingly deregulated with age. Since both of these factors are autocrine, an important doubt arises: is NSC deregulation a consequence of niche unbalance? Or are increasingly malfunctioning NSCs the thriving force that disrupts neurogenesis? Regardless of the cause, it seems that Wnts and PTN partially mediate the age-associated decrease in neurogenesis. In addition to playing a role in brain homeostasis, PTN is also considered of playing a role in disease. Finally, a study found that PTN signaling ameliorates impaired neurogenesis in AD and it is necessary for the neurogenic effects of an enriched environment [93].

## Conclusion and Future Perspectives

There is a long way for NSCs to mature and integrate into the established brain network. In the first stages of their life, NSCs remain quiescent and, when the appropriate conditions are reunited, they become active and proliferate either symmetrically or asymmetrically. After an asymmetrical division, NSCs originate NPCs that either proliferate or further differentiate into neurons or glial cells. All of those steps imply decisions that are dependent on a variety of factors from different sources that in the end, converge through signaling pathways with the same downstream effectors. Interestingly, the activity of these downstream effectors is often dependent on the delicate balance between stimulatory and inhibitory signals.

In this context, the Akt pathway emerges as a key player in signal integration among NSCs, being often responsible for the delicate choice between proliferation and quiescence or proliferation and differentiation. Strikingly, the Akt pathway is shown to be responsible, or partially responsible, for the actions of half of the neurogenic signals described here. One example of such interaction occurs between Wnts and the IGF-II signaling. Upon activation of the Wnt pathway, there is β-catenin accumulation in the cytoplasm which upregulates *Akt* expression. Interestingly, Akt is a known promoter of β-catenin, suggesting that with adequate conditions, these two pro-proliferative pathways share positive feedback loops that push the NSCs toward proliferation. Other known activators of the Akt pathway are VEGF, which has a similar role in neurogenesis, IGF-II and PTN.

Under homeostatic conditions, Akt signaling appears to be continuously inhibited by phosphatase and tensin homolog (PTEN), otherwise, NSCs would become active and the NSC pool eventually depleted [94]. These effects seem to be mediated by the downstream activation of mTOR and by inhibition of the pro-quiescence transcription factor FOXO 3 [95, 96]. In addition to PTEN, MCFD2 also inhibits Akt activation playing an important role as a “safety mechanism” to prevent excessive NSC activation. However, it is important to note that the signal integration of the Akt pathway does not exclusively rely on shared downstream effectors. Indeed, the Akt pathway was found responsible for the proliferative effects of the sonic hedgehog (Shh) by an alternative mechanism. Unlike other mitogens that act through this pathway, the period between Shh signal stimulation and increased Akt activation suggests Shh does not directly activate Akt [97]. In contrast, Shh appears to increase the production of other autocrine factors that, ultimately, activate the Akt pathway, e.g. platelet-derived growth factor receptor [98] and IGF-II [99]. This observation added therefore a layer of complexity to the possible molecular interactions between neurogenic signals. However, it also indicated that every signal is crucial to the regulation of NSCs since the deregulation of a single factor could affect the entire signaling network.

On the other hand, it thus appears that, if a vast number of factors lead to NSC activation and proliferation, other types of factors act on pro-quiescence pathways and produce the opposite result. One of those factors is the BMP family of proteins. When stimulated, these proteins increase the expression of the anti-differentiation Id proteins [100] that stabilize the expression of the Hes proteins [101]. In fact, these proteins are essential for the regulation of NSC quiescence since they are the effectors of both the BMP and Notch quiescence pathways. Their cellular level dictates the choice between quiescence or proliferation after Notch pathway activation. High levels of Hes proteins inhibit the expression of the pro-proliferation gene *Ascl1* while low, or oscillating, levels of Hes lead to unsynchronized oscillations of Ascl1 that result in NSC proliferation [102]. All in all, the BMPs and Notch pathways are considered key regulators of NSC quiescence. Unlike most of the factors described here, they are two of the few that actively prevent NSC activation and contribute to the maintenance of an adequate NSC pool. Curiously, in the SVZ, type C and A cells express Notch ligands that are responsible for determining the rate of neurogenesis [18]. In addition to BMPs and Notch, the homologue of the Drosophila tailless gene (TLX) factor also influences Ascl1 actions, promoting its expression and, as a consequence, functioning as a proliferative and anti-differentiation factor.

When the appropriate conditions are present, a quiescent NSC becomes active and proliferates, originating several NPC cells. Upon maturation and migration, these cells integrate into pre-existing neural networks either at the OB or at the DG in a process formally described as neurogenesis. This process declines with age in both humans and rodents [103]. Interestingly, the age-related decline in neurogenesis occurs in parallel with poorer behavioral pattern separation (the process of distinguishing two very similar inputs) performance, suggesting a possible relation between worst cognitive performance and diminished hippocampal neurogenesis. Although the exact mechanism is yet to be elucidated, several studies correlated hippocampal neurogenesis inhibition with cognitive deficits, namely impaired spatial memory consolidation and context-dependent memory [62, 104, 105]. One promising hypothesis suggests that newly-born neurons are key players in the relation between neurogenesis and healthy cognition. Intriguingly, these neurons are more responsive than their mature counterparts, which theoretically could render them ineffective in pattern separation, a function that requires accurate activation of neurons. However, two studies revealed that these newborn neurons reduce the basal DG activity making it respond more accurately to stimuli and less responsive to noise [106, 107]. Through this process, NSCs continuously monitor systemic cues which are often affected by processes like physical exercise, diet and even emotional states, and relay them to the neurogenic niche ultimately affecting the long-term homeostasis of the brain. In fact, this mechanism can be responsible for the positive correlation that exists between healthy diets and exercise and increased cognitive performance.

Apart from its key role in consolidating pattern separation, hippocampal neurogenesis is also pivotal in the consolidation of engrams. An engram, or a memory trace, can be defined as an actual cellular representation of an experience. For example, in fear-conditioning tasks, when rats are exposed to a context before an aversive event, a group of cells in the hippocampus is activated. Every time the animal is exposed to the same context, the same cellular pattern becomes active, and the animal displays fear and anxiety-like behavior. These engrams can be associated with fear and reward, and their initial association can be changed among new experiences. Likewise, with pattern separation, neurogenesis silencing impairs engram activation [108]. Indeed neurogenesis is responsible for memory clearance in the hippocampus, a process that eliminates conflicting memories, reduces memory interference and allows better incorporation of newly formed memories [109]. Through this process, SGZ neurogenesis greatly contributes to the association of a context to an outcome, one of the most studied roles of the hippocampus.

As depicted in Table 1, this review sheds light on how different autocrine and paracrine signals influence NSC behavior. Particularly, it highlighted the balance of several pathways outcomes to determine the NSC decision to either activate, proliferate, or differentiate. Nonetheless, this grid-like activity can be considered a safety mechanism to prevent possible catastrophic consequences from the imbalance of a single factor.

**Table 1:** Autocrine and paracrine effects of NSC endogenous factors

Signal	Niche	Type of Study	Mode of Action	Effects	References
Wnts	SGZ/SVZ	In vivo/ in vitro	Autocrine	NSC proliferation and differentiation	[24]
Glutamate	SGZ	In vivo/in vitro	Paracrine	NSC self-renewal	[41]
GABA	SVZ	In vivo	Paracrine	Repression of NSC self-renewal	[44, 45]
DBI	SVZ	In vivo	Paracrine	NSC proliferation	[46, 47]
MCFD2	SGZ	In vitro	Autocrine	NSC survival/Stemness	[53, 54]
IGF-II	SGZ	In vivo	Autocrine	NSC self-renewal/Stemness	[62]
VEGF	SGZ	In vivo	Autocrine	NSC self-renewal/Stemness	[72]
BMP’s	SGZ/SVZ	In vivo/in vitro	Autocrine	Quiescence in NSCs	[77, 78]
PTN	SGZ	In vivo	Autocrine	Neural arborization and maturation of NSCs	[92]

Despite the research that has been developed during the past years, there is still a lot to be understood in terms of the homeostatic mechanisms of neuroregeneration. One example is the influence of microRNAs and other molecules, such as metabolites, present in the NSC surroundings. A better understanding of these additional layers of factors will allow a better characterization of the neurogenic niches, and eventually the development of innovative NSC-based therapies. Overall, NSCs act as protectors and guardians of the brain, monitoring and responding to signals to maintain its health and function.

## Data Availability

This article will be accessed online upon publication.
